# Increasing malaria hospital admissions in Uganda between 1999 and 2009

**DOI:** 10.1186/1741-7015-9-37

**Published:** 2011-04-13

**Authors:** Emelda A Okiro, David Bitira, Gladys Mbabazi, Arthur Mpimbaza, Victor A Alegana, Ambrose O Talisuna, Robert W Snow

**Affiliations:** 1 Malaria Public Health & Epidemiology Group, Centre for Geographic Medicine Research - Coast, Kenya Medical Research Institute/Wellcome Trust Research Programme, Nairobi, Kenya; 2 Centre for Tropical Medicine, Nuffield Department of Clinical Medicine, University of Oxford, CCVTM, Oxford, UK; 3 International HIV/AIDS Alliance-Uganda, Kampala, Uganda; 4 Uganda Malaria Surveillance Project, Kampala, Uganda; 5 IDRC-U, Kampala, Uganda

## Abstract

**Background:**

Some areas of Africa are witnessing a malaria transition, in part due to escalated international donor support and intervention coverage. Areas where declining malaria rates have been observed are largely characterized by relatively low baseline transmission intensity and rapid scaling of interventions. Less well described are changing patterns of malaria burden in areas of high parasite transmission and slower increases in control and treatment access.

**Methods:**

Uganda is a country predominantly characterized by intense, perennial malaria transmission. Monthly pediatric admission data from five Ugandan hospitals and their catchments have been assembled retrospectively across 11 years from January 1999 to December 2009. Malaria admission rates adjusted for changes in population density within defined catchment areas were computed across three time periods that correspond to periods where intervention coverage data exist and different treatment and prevention policies were operational. Time series models were developed adjusting for variations in rainfall and hospital use to examine changes in malaria hospitalization over 132 months. The temporal changes in factors that might explain changes in disease incidence were qualitatively examined sequentially for each hospital setting and compared between hospital settings

**Results:**

In four out of five sites there was a significant increase in malaria admission rates. Results from time series models indicate a significant month-to-month increase in the mean malaria admission rates at four hospitals (trend *P *< 0.001). At all hospitals malaria admissions had increased from 1999 by 47% to 350%. Observed changes in intervention coverage within the catchments of each hospital showed a change in insecticide-treated net coverage from <1% in 2000 to 33% by 2009 but accompanied by increases in access to nationally recommended drugs at only two of the five hospital areas studied.

**Conclusions:**

The declining malaria disease burden in some parts of Africa is not a universal phenomena across the continent. Despite moderate increases in the coverage of measures to reduce infection and disease without significant coincidental increasing access to effective medicines to treat disease may not lead to severe disease burden reductions in high transmission areas of Africa. More data is needed from a wider range of malaria settings to provide an honest tracking progress of the impact of scaled intervention coverage in Africa.

## Background

Efforts to reduce the burden of malaria in Africa have intensified through the scaled delivery of effective tools for malaria prevention and disease management, facilitated by increased donor funding and a commitment by national governments and their partners [1]. There have been a number of reports of declining malaria morbidity and mortality, in some cases directly or plausibly linked to the scaling up of control efforts [2-13]. However, many of these reports are from areas with relatively low baseline malaria transmission intensity (South Africa; large parts of Zambia, Ethiopia, Eritrea, Rwanda), island communities (Zanzibar; Bioko, São Tomé and Príncipe), or represent areas with a long history of malaria research and surveillance systems (Kilifi, Kenya and The Gambia). It is striking how little data have been published from areas in Africa characterized by high transmission and less intense rapid scaling of intervention coverage.

In Kenya, following a nationally representative review of pediatric malaria admissions to hospitals between 1999 and 2009 we noted that the declining incidence of malaria admission was not a universal observation across the country [8] despite relatively consistent temporal increases in intervention coverage between sites [9]. Kenyan hospitals where declining malaria admission rates were far less notable, or showed evidence of increasing with time, were located in areas with higher malaria transmission intensity [9]. To extend the empirical observations of possible temporal disease burden changes in Africa across a wider range of transmission conditions and intervention coverage success, we present data assembled from five hospital sites in Uganda between 1999 and 2009 to improve our understanding of the proposed epidemiological transition in Africa.

## Methods

### Ugandan malaria context

Uganda is a landlocked country traversed and bordered by large inland water bodies. *Plasmodium falciparum *transmission is intense and largely perennial and 1 of the 15 highest transmission intensity countries worldwide [14]. At 7 sites across Uganda entomological inoculation rates (EIR) from locally dominant vectors, mostly *Anopheles gambiae *s.s., ranged from 4 to over 1,500 infectious bites per person per annum [15], the latter recorded at Apac represents the highest published estimate of EIR in Africa. Areas with lower transmission intensity are located along the high altitude areas bordering Democratic Republic of Congo and Rwanda, the slopes of Mount Elgon in the East, arid areas in the North and the central areas of the urban extents of Kampala [16-18]. The national estimate of age-under-5 mortality has been modeled to be approximately 145 per 1,000 live births for 2000 and predicted to have declined to 117 per 1,000 live births by 2010 [19,20].

In 2001 a 5-year national malaria strategy was launched, which had as its goal to 'prevent and control morbidity and mortality and to minimize social effects and economic losses attributable to malaria in the country' [21]. The strategy included one measurable health outcome of reducing by 3% malaria case fatalities by 2005 and included a process outcome of ensuring that at least 50% of children are protected by an insecticide-treated net (ITN) by 2005 [21]. In 2005 the national strategy was rewritten, retaining a similar goal but explicitly aiming to contribute to reducing age-under-5 mortality from 152 to 103 per 1,000 live births with the number of under 5 s protected by an ITN increased to 85% by 2010 [18].

Since 2004, three successful applications to the Global Fund have resulted in approximately $200 million committed and $146 million disbursed to Uganda to support a wide range of commodity needs and implementation activities by the end of 2009 [22]. Between 2005 and 2009 the President's Malaria Initiative (PMI) provided $110 million to promote better coverage of insecticide treated nets (ITN) though free distribution (since 2007), mounting indoor-residual house-spraying activities in selected districts and internally displaced camp populations, the nationwide improvement of malaria case management and drug management supply and improved training for antenatal care providers to support intermittent presumptive treatment [23]. UNICEF and bilateral donors including the US pre-PMI, the UK, Belgium and Canada have all contributed approximately $10.6 million since 2004 for malaria control activities in Uganda [24].

Despite documented widespread chloroquine (CQ) resistance across the East African region, including Uganda, by 2001 [25,26], this drug was widely used in combination with sulfadoxine-pyrimethamine (SP) as first-line therapy in government clinics and promoted as a national strategy to provide a home-based management package (Homapak^®^) between 2003 and 2005 [27-29]. Both CQ and SP clinical failure rates reached exceptionally high levels by 2005 [30,31] and the policy for malaria case management was finally changed in April 2005. In 2006 the national malaria treatment guidelines were revised to support the introduction of artemether-lumefanthrine (AL) as first line therapy [32-34] and effectively rolled out to all clinics by April 2006. However, AL funding, tendering and drug management supply to the periphery has been less than perfect since 2004 [35,36]. Between May and August 2007 it was shown that 75% of health facilities in four districts had experienced a stock out of one of the four AL packs, including 34% of facilities that had stocked out of all treatment packs at least once simultaneously in the preceding 6 months and 13% of facilities did not have any AL packs in stock on the day of survey [36]. The early implementation problems may in part explain why only 3% of fevers among children reported treatment with AL in 2006 [37]. However, in 2008, between September and October 58% of the Public Health Facility had experienced a stock out of at least one of the four AL packs in the 3 months prior to survey and on the survey day 17% were out of stock of at least one of the four AL packs [38]. Private, retail sector availability of AL or other artemisinin-based products is uncommon; a study of 1,398 retail outlets across 38 districts in 2008 found no routine retail outlet stocked AL or any artemisinin-based combination therapies (ACT) and only 4.3% of drug stores stocked AL [38]. However in 2009 two sample household surveys showed that 21% [39] and 23% [40] of childhood fevers were treated with AL at any time during the febrile illness occurring within the last 2 weeks.

In 2000 less than 1% of Ugandan children were protected by an ITN [41,42]. Between 2001 and 2003 there was a slow increase in activities to socially market or use voucher schemes to promote ITN distribution, however, ITN coverage among children only marginally increased to 9.7% during a national household sample survey undertaken in 2006 [37]. From 2007, using PMI support, free mass distribution campaigns and provision free of charge of ITN to antenatal clinics has resulted in an increase in children protected by ITN as evidenced by the results of the most recent national household sample survey undertaken in 2009 that showed ITN use among children had risen to 32.8% [40]. Indoor residual house spraying (IRS) activities have targeted areas of unstable transmission including internally displaced persons and refugee camps. Before 2006 IRS activities were limited. The first large-scale pilot IRS campaign supported by PMI was introduced in the epidemic-prone highland district of Kabale in 2006 and was subsequently repeated in 2007 and 2008 this time including Kanungu district [43] and extended to several districts in the North. From March to April 2008, pilot IRS campaigns with DDT were carried out in the high transmission districts of Oyam and Apac and of the households identified for spraying, it was reported that the campaign covered over 90% of the targeted population [44].

### Hospital selection

**Figure 1:**
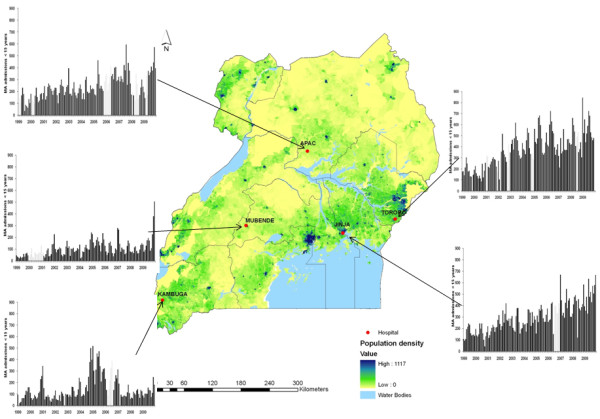
**Monthly cases of malaria admission**. A 100 × 100 m human population density map developed from fine resolution satellite imagery, land cover and available information on human population counts from the highest resolution census administration boundaries showing the catchment areas around each hospital [47,48]. Graph panels show monthly cases of malaria admissions observed (black bars) and imputed (gray bars) across the 11-year period.

There are an estimated 65 government-supported hospitals (2 national referral hospitals, 13 regional referral hospitals and 50 general hospitals) providing inpatient pediatric care services across Uganda. For this study five relatively high pediatric admission burden hospitals were identified in consultation with the Ministry of Health to reflect the diversity of malaria transmission across Uganda [15,45] and were most likely to provide records of admissions over the last 11 years (Figure 1). The hospital sites were representative of the national hospital distribution and previous drug sensitivity district selections by the Ministry of Health [25], with the exception that no hospital was selected in the concentrated urban population living in Kampala. Mubende hospital is located in a moderate-to-high transmission area of the Central 2 region; Jinja is located in the East Central region bordering Lake Victoria and represents an area of historically moderate transmission and the hospital serves a higher urban community than the other four hospitals; Kambuga hospital in Kanungu district serves a population that borders the Democratic Republic of Congo in the South Western Region and characterized by low, highland transmission intensity; Tororo is situated in the Mid-Eastern region on the Kenyan border and supports high perennial transmission; and finally Apac hospital is located centrally in the Mid-Northern region and experiences exceptionally intense transmission.

### Pediatric admission data

Pediatric ward inpatient registers at all hospitals were identified for most months from January 1999 to December 2009. Each admission entry in the registers was recorded on a tally sheet indicating the month of admission and whether an admission diagnosis of malaria had been defined for the child or whether the admission diagnosis did not include an indication of malaria.

Exact dates of birth were not available for all admissions and we have assumed all pediatric admissions were aged between 0 and <15 years. Individual register entries were not reconciled with patient notes and we have assumed that the admission diagnosis remained the diagnosis upon which each admission was managed clinically. Slide confirmed malaria diagnoses at admission were not universally available within and between hospital sites for the surveillance period and this continues to pose a significant information gap [46]. Therefore our working definitions of 'malaria' were patients admitted with a diagnosis of malaria, probably managed clinically as malaria during their admission but without documented parasitological confirmation.

For each possible 132-month series at each hospital, data were missing for 6 months (5%) at Jinja, 8 months (6%) at Kambuga, 13 months (10%) at Tororo, 14 months (11%) at Mubende and 21 months (16%) at Apac because registers were missing or torn. The missing data were handled using the 'ice' command in Stata (v.11.1; Stata, College Station, TX, USA) by creating multiple imputed data sets for missing values using other existing variables and prior information (the number of malaria and non-malaria deaths or admission as appropriate) to create an imputed value for each of the missing months. The same algorithm was used across all sites with all missing data imputations performed per sites (that is, using data from that site only).

It is important to standardize changing admission rates with time between hospitals with respect to the catchment population and natural growth in populations served by each hospital. Defining precise catchment areas is non-trivial and we have previously used addresses of pediatric admissions to explore the spatial nature and form of catchment areas at 17 Kenyan hospitals [8]. Similar information and detailed, high-resolution settlement mapping were not available in Uganda. We have therefore used an average radial distance of 30 km (that captured over 90% of admissions in Kenya) for the purposes of standardizing the catchment areas of the five Ugandan hospital sites. With the exception of Jinja, which is a regional referral hospital, all the others are district hospitals and the defined catchment area are inclusive of the subcounties where health workers in each hospital say majority of their patients originate. This radial boundary was used in combination with a 100 × 100 m human population density map developed from fine resolution satellite imagery, land cover and available information on human population counts from the highest resolution census administration boundaries [47,48]. Population counts within a 30 km radius of each hospital were extracted in ARCGIS 9.2 (ESRI, Inc., Redland, CA, USA) for the Ugandan census date of 2002. Other methods have been used elsewhere [8] to define catchment populations but depend on the availability of reliable data on residential location of admitted patients. There is remarkably little published data on distances travelled for severe illness requiring hospitalization and this is an area that demands further investigation. Meanwhile, 30 km has been shown to be a reasoned catchment distance for hospitals in Kenya and therefore elected for use here in Uganda. District-specific population age structures and natural annualized growth rates [49] were used to project forwards and backwards to provide an estimated 0-14-year-old population estimate for each of the survey years 1999 through to 2009. Malaria and non-malaria incidence was computed using the estimated children aged 0-14 years resident in the hospital catchment area. Confidence intervals around each annualized admission rate per 1,000 children aged 0-14 years were computed using the Poisson distributions [50].

In addition we have collapsed average annualized admission incidence rates across three key time periods: (a) a period that corresponds to the widespread use of CQ-SP, little progress toward scaled ITN/IRS coverage and limited external donor financial support (1999-2003); (b) a period of transition to AL implementation, moderate increases in ITN coverage and improved external donor assistance (2004-2006); and finally (c) a period that witnessed a large increase in external funding from The Global Fund to Fight AIDS, Tuberculosis and Malaria (GFATM) and PMI, a concerted effort to provide free ITN distribution including mass campaigns in 2007, the introduction of IRS in selected districts (including Apac and Kanungu) and an established single recommendation for AL and a withdrawal of CQ-SP (2007-2009). Furthermore, data on the coverage of interventions during these periods are available from geolocated cluster sample survey data from national surveys to explore the plausible correspondence between changing prevention and case management strategies and disease incidence between time intervals and between hospital settings (Table 1).

**Table 1:** Hospitalization and intervention characteristics of selected study regions

Characteristic	District (region)				
	
	Kambugu Hospital (South Western)	Mubende Hospital (Central 2)	Jinja Hospital (East Central)	Tororo Hospital (Mid-Eastern)	Apac Hospital (Mid-Northern)
Number of beds	135	100	431	285	100

Projected population <15 years in 30 km radial catchment:					

1999	235,448	116,802	398,969	310,100	116,910

2009	280,321	159,316	522,751	413,633	166,559

Average annual rainfall 1999-2009, mm	1,009	1,062	1,359	1,553	1,471

Malaria admission rate per 1,000 catchment of 0-14-year-old population per year 1999-2009 (95% CI), total malaria records	6.85 (6.76 to 6.95), 19,398	9.20 (9.04 to 9.35), 13,860	8.71 (8.62 to 8.80), 39,355	12.49 (12.38 to 12.60), 49,413	20.30 (20.07 to 20.52), 31,351

Non-malaria admission rate per 1,000 catchment of 0-14-year-old population per year 1999-2009 (95% CI), total non-malaria records	1.41 (1.37 to 1.46), 7,607	5.05 (4.94 to 5.16), 7,607	4.34 (4.28 to 4.40), 19,622	2.93 (2.88 to 2.98), 11,595	11.63 (11.46 to 11.81), 17,970

Intervention coverage:^a^					

UDHS 2000/2001					

Percentage of children under 5 years with fever treated with recommended first-line drug CQ (n)	10.4 (316)	10.2 (206)	25.0 (356)	17.6 (278)	9.6 (187)

Percentage ITN coverage in children aged <5 years^b ^(n)	0.15 (1374)	0.92 (649)	0.36 (845)	0.73 (683)	0.50 (399)

UDHS 2006					

Percentage of children under 5 years with fever treated with recommended first-line drug CQ + SP (n)	19.5 (205)	8.3 (240)	9.6 (374)	8.4 (346)	16.8 (499)

Percentage ITN coverage in children aged <5 years^b ^(n)	7.1 (917)	4.2 (793)	5.0 (925)	6.5 (799)	23.1 (1063)

UMIS 2009					

Percentage of children under 5 years with fever treated with recommended first-line drug AL (n)	10.0 (106)	18.0 (158)	13.4 (322)	16.6 (130)	40.8 (361)

Percentage ITN coverage in children aged <5 years^b ^(n)	32.6 (487)	11.3 (352)	18.9 (564)	41.8 (479)	41.8 (568)

Proportion of households sprayed in last 12 months (n)	1.8 (705)	4.6 (439)	0.4 (557)	0.6 (530)	31.6 (552)

Transmission intensity at end of surveillance period 2009					

Parasite rate among children aged 1-59 months November-December 2009 (n)	11.6% (460)	50.7% (344)	56.3% (538)	37.5% (472)	62.5% (554)

^a^Individual level data from the Uganda Malaria Indicator Survey (UMIS) undertaken between November and December 2009 was not available at the time of publication thus we have resorted to reporting regional estimates. The sample was stratified into nine survey regions across the country, plus Kampala. Individual-level data from the Uganda Demographic and Health Surveys (UDHS) undertaken in September-March 2000/2001, May-October 2006 data were reconstructed from empirical data available at http://www.measuredhs.org/ and extracted to correspond to intervention coverage within the regions used in the MIS 2009.

^b^Insecticide-treated net (ITN) coverage was defined as the percentage of children aged under 5 who slept under an ITN last night and a treated net defined as one that was long lasting or had been soaked or dipped in insecticide in last 6 months.

AL = artemether-lumefanthrine; CQ = chloroquine; SP = sulfadoxine-pyrimethamine;.

### Time series analysis

To examine the long-term trends in admission rates we used monthly malaria admission data, standardized for under 15 population densities within each catchment areas, with moving average smoothing methods to identify any long-term trend signals within each temporal 11-year series while filtering out short-term annual fluctuations and random variation. The smoothing technique achieves this by replacing each element of the time series by *n *neighboring elements, where *n *is the width of the smoothing 'window' equal to 12 months. We employed a centered moving average including six observations before and five after the time point and vice versa, and taking an average value of this to compute the centered average. The ARMAX ('AutoRegressive Moving Average model with eXogenous inputs') model was then applied [51], which is an autoregressive model of the empirical current value of the series against one or more prior values combined with a moving average of the current value modeled against the white noise (random shocks) of one or more prior values and includes explanatory variables. The established explanatory variables for malaria trend analysis included rainfall in the preceding months (at different significant timelags), assembled from meteorological stations located close to each hospital, and changes in service use (captured by non-malaria admission rates) resulting in a predicted or smoothed malaria admission rate per month for each hospital site over the period 1999 to 2009. Correlations between rainfall at different timelags and malaria cases were assessed before including significant variables in the final ARMAX model. Before specifying the ARMAX model, tests and diagnostics were performed in the estimation of the models. Monthly malaria rates were tested for stationarity using the augmented Dickey-Fuller test [52] with a lag of 12 months. Model diagnostics and selection criteria were used to determine the most parsimonious model. Two goodness-of-fit criteria were used to guide model selection: Akaike information criterion (AIC) and Schwartz's Bayesian information criterion (BIC). The models with the lowest AIC and BIC were finally selected. All analysis was undertaken using Stata V. 11.0.

## Results

### Descriptions of hospital data

Information was assembled on 146,473 (72%) malaria admissions and 58,115 (28%) admissions where a diagnosis of malaria was not made at admission among children aged less than 15 years of age at the 5 hospitals over 11 years (January 1999-December 2009). Corrected malaria admission rates standardized for population estimates within each hospital catchment over the entire period varied between hospital sites, ranging from 6.9 per 1,000 children 0-14 years per year in Kambuga to 20.3 per 1,000 children per year in Apac (Table 1). These differences between hospitals were similar between the three important time periods and none of the annualized period incidence estimates were significantly different between time periods within each hospital site with the exception of a higher incidence at Kambuga during the second period (2004-2006) and a general tendency at all sites for the malaria admission rates to be higher in the last period, 2007-2009, compared to the first period 1999-2003 (Table 2). Non-malaria admission rates across the three time periods demonstrated either no changes at Mubende and Kambuga, a slight increase at Tororo and marginal declines at Jinja and Apac. With the exception of one period in Jinja, none of these differences were significant with overlapping confidence intervals in all estimated time period rates (Table 2).

**Table 2:** Temporally aggregated pediatric admission data for malaria each of the five hospitals between 1999-2003, 2004-2006 and 2007-2009 expressed per 1,000 children aged 0-14 years at risk per annum and 95% confidence intervals computed using a Poisson distribution

Hospital location	Kambuga	Mubende	Jinja	Tororo	Apac
Average annual malaria admission rate 1999-2003 (95% CI), total number of malaria admissions	4.79 (4.52 to 5.07), 5,846	7.80 (6.62 to 7.56), 4,448	5.93 (5.70 to 6.16), 12,570	9.56 (9.23 to 9.89), 15,899	17.18 (16.47 to 17.92), 10,879

Average annual malaria admission rate 2004-2006 (95% CI), total number of malaria admissions	11.95 (11.54 to 12.37), 9,372	10.39 (9.86 to 10.93), 4,374	7.89 (7.63 to 8.14), 11,114	14.74 (14.35 to 15.14), 16,332	22.04 (21.28 to 22.82), 9,602

Average annual malaria admission rate 2007-2009 (95% CI), total number of malaria admissions	5.06 (4.80 to 5.33), 4,180	10.84 (10.33 to 11.36), 5,038	10.25 (9.98 to 10.53), 15,671	14.20 (13.84 to 14.58), 17,182	22.56 (21.84 to 23.30), 10,870

Average annual non-malaria admission rate 1999-2003 (95% CI), total number of non-malaria admissions	1.50 (1.35 to 1.66), 1,823	4.82 (4.44 to 5.23), 3,000	4.69 (4.49 to 4.90), 9,880	2.89 (2.71 to 3.08), 4,729	11.89 (11.27 to 12.47), 5,328

Average annual non-malaria admission rate 2004-2006 (95% CI), total number of non-malaria admissions	1.50 (1.35 to 1.65), 1,176	5.08 (4.71 to 5.47), 2,142	3.25 (3.09 to 3.41), 4,568	2.32 (2.16 to 2.48), 2,570	10.67 (11.66 to 12.80), 5,328

Average annual non-malaria admission rate 2007-2009 (95% CI), total number of non-malaria admissions	1.21 (1.08 to 1.34), 998	5.31 (4.96 to 5.69), 2,465	3.38 (3.22 to 3.54), 5,174	3.59 (3.41 to 3.78), 4,296	10.67 (10.18 to 11.18), 5,119

**Figure 2:**
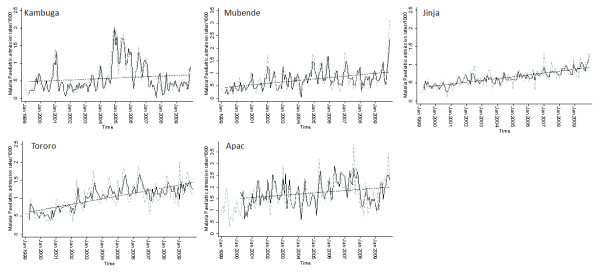
**Model predictions of malaria hospitalization rates**. Plots of model predictions of malaria hospitalization rates controlling for lagged rainfall and non-malaria cases and controlling for autoregressive and moving average effects (solid black line) and observed malaria hospitalization rates (green dotted line). Fitted lines illustrate the linear trends from model predictions (dashed line).

To examine the long-term temporal signals within the monthly time series several model forms were explored. The predictions from the best fitting model that adjusted for non-malaria admission rates and rainfall at different lags while controlling for autoregressive and moving average effect within the data are shown in Figure 2. We included linear trend lines fitted to estimated average monthly changes in malaria admissions. The relationship between malaria admissions by month show increases across all five sites, although this appears less clear in Kambuga where there were exceptional peaks in malaria admission rates in 2005/2006. Significant (*P *< 0.001) upward trends in malaria admission rates were observed in Mubende, Jinja, Tororo and Apac over the study period (Figure 2). By 2009 the relative proportional increase in malaria admission cases from 1999 was greatest in Mubende (+350%) with a significant peak in 2009, but important increases were seen in all other sites when compared to 1999 at Jinja (+200%), Tororo (+200%), Apac (+188%) and Kambuga (+47%) (Figure 1).

## Discussion

Across all five hospital sites investigated, malaria admission rates have increased or remained unchanged since the period of prescaled intervention and funding (1999-2003) through a period when Uganda had received significant overseas donor support (2007-2009). Conversely non-malaria pediatric admissions remained relatively constant across all three observation periods 1999-2003, 2004-2006 and 2007-2009 (Table 2). Analysis of monthly time series of malaria admission rates using autoregressive models adjusting for external factors show that there was a significant increase in pediatric admissions from January 1999 to December 2009 (Figure 2; all linear fits *P *< 0.001) in four of the five sites. The net, proportional increases across all sites in malaria admissions from the beginning of 1999 to the end of 2009 ranged from 47% to over 350% depending on the hospital site. For example in Apac the proportion of malaria admissions was 54% in 1999 and increased to 70% by 2009 while in Jinja the proportion of malaria admissions increased from 49% to 73%.

We have adjusted for a number of factors that might influence a rising temporal hospital presentation of malaria including intrinsic population growth among the pediatric population's served by each hospital, between yearly seasonal variations in rainfall patterns and a proxy for overall hospital use through the adjustment for admissions not diagnosed as malaria. It seems therefore entirely plausible that malaria admissions have risen in four of the five sites included in the analysis. The site located in an area of lower transmission intensity experienced a large 'epidemic' period of malaria between 2005 and 2006 that distorted the linear time series analysis, however even at Kambugu the average malaria admission incidence between 1999-2003 was not significantly different from the period after large funding and implementation activities 2007-2009 (Table 2).

We have not been able to adjust for the proportion of true malaria diagnoses among children admitted to each hospital or changing diagnostic practices that may create a systematic bias in observed trends. This presents a perennial problem for all retrospective hospital reviews where parasitological diagnoses are not available for systematic review or data linkage. Nevertheless similar reviews of admission records, with similar caveats, have been used repeatedly to support claims of declining incidence of malaria admissions [7,8,10,11,53,54] and therefore should be viewed with equivalent validity for increasing incidence. Changing practices and support to improve the diagnosis of malaria with time are likely to have resulted in fewer false positives and therefore one might expect this to support an observation of declining malaria diagnosed admissions.

Despite the incomplete nature of centrally reported hospital admission data, the national Health Information Systems (HMIS) of the Ugandan Ministry of Health suggest that the malaria case burden in public health facilities has increased from 3.5 million in 2000 to 10.7 million in 2004 and risen further by 2008 to over 12.2 million cases [46]. Although HMIS data are plagued by gaps and changes in reporting rates between and within years, these parallel data circumstantially support a rising, rather than declining, trend in malaria burden in Uganda. User fees in government health facilities were abolished in 2000, thus potentially creating a systematic bias in observed trends but the increasing trends remained significant after adjusting for non-malaria cases used here as a proxy for changes in hospital usage.

Uganda has been granted almost $267 million in overseas donor assistance since 2002 and this has translated into an average annual external commitment to malaria funding of approximately $2 per person at risk in Uganda [24], within $0.5 of requirements predicted to provide sufficient funding for a complete package of intervention delivery [55]. National coverage with essential interventions remains below international targets set 10 years ago in Abuja, Nigeria [56] and those set by national malaria strategies launched in 2001 and 2005 covering 5-year periods, respectively [18,21]. By 2009 the proportion of children sleeping under an ITN was only 33% and less than a quarter of children with a febrile illness had access to the nationally recommended first-line treatment. However, there are important differences in intervention coverage between districts that are served by the five hospitals included in this study (Table 1). At Apac and Tororo, two focus districts for PMI support, ITN coverage among children exceeded 40% by 2009 while it was less than 20% in Mubende and Jinja districts. However it is important to consider all facets of malaria control and while ITN and IRS coverage has improved with time, the proportion of febrile children who access efficacious, recommended antimalarial drugs has declined. In 2000, access to CQ was equivalent to the access reported in 2009 to AL in three of the five study areas shown in Table 1. However over 40% of febrile children living in the area around Apac were treated with AL in 2009 while all other districts only managed to report less than 20% use of AL in 2009 [40]. Despite these differences and the combined efforts to provide IRS with PMI support in Apac, increasing intervention coverage has yet to translate into observed reductions in hospitalization. Uganda is still characterized by predominantly high malaria transmission intensity, as judged by the parasite prevalence recorded in children aged less than 5 years. By the end of the surveillance period transmission intensity remained high across Mubende, Jinja, Tororo and notably high at Apac but low in Kambugu (Table 1).

It is important to begin to define why large-scale donor support has not translated into significant intervention coverage. There were concerns about the transparent use of GFATM funding in Uganda that led to suspension of funding in August 2005 and led to the interruption in funding flows and planned activities [57]. Procurement and provision of effective medicines through responsive drug management supply chains has been the subject of international concern around tendering processes [35,58,59] and documented shortages of malaria medicines in the periphery [36,38]. In the 1980s and 1990s many settings witnessed an increase in malaria cases as a result of increasing resistance and declining efficacy of antimalarial drugs. This status quo is perpetuated today where despite the existence of more efficacious drugs these are far from universally available resulting in an overall population effectiveness of effective treatment that could perpetuate a continued rise in disease incidence despite only moderate increases in ITN coverage. The preferred options for delivering ITN remained anchored in client payment systems for many years, albeit subsidized, but not free until late in 2007. Mass ITN distribution campaigns however have not raised coverage to effective levels anticipated to impact upon transmission [60]. The current level of ITN coverage is inadequate to significantly impact on disease incidence given the levels of high transmission intensity that characterize Uganda [60]. Additionally, six different national malaria control program managers have been appointed over the last 10 years and this has inevitably resulted in a loss of institutional memory and consistent stewardship. Despite these shortfalls in malaria control implementation since 2004 the Ugandan national malaria strategy for 2011-2015 has as its vision that 'malaria will no longer be the major cause of illness and death in Uganda and families will have universal access to malaria prevention as well as treatment by 2015' supported by a mission statement that states that the Ministry and its partners will 'reduce the level of malaria infection and consequent malaria death in Uganda by 75% by the year 2015, and to sustain that improved level of control to 2020'.

There is a general sense that all of Africa is witnessing an epidemiological transition that is a direct result of international donor support [61]. This position has been supported by recent reports of a declining malaria incidence across a number of sites in sub-Saharan Africa [2-8,10-13,53,62,63] and modeled predictions of the malaria-specific mortality effect size of increased intervention coverage [61,64]. However, as noted in a recent review of the evidence, that still supported the view that scaled intervention has contributed to declining malaria incidence across Africa, there remains a publication bias in favor of 'good news stories' [65] from areas of traditionally low transmission intensity and rapid, concerted scaling of disease prevention and management strategies. The modeling exercises that use the Lives Saved Tool (LiST) [61,64,66,67] make various assumptions about the impact size derived from randomized controlled trials [68] on a fraction of age-under-5 mortality presumed to be malaria from verbal autopsy studies [69]. The LiST model does not account for variations in either intervention effect size or malaria burden by malaria transmission intensity. The predicted impacts on under-5 mortality are also based on single intervention effect sizes, for example the expected numbers of deaths averted defined by the protective efficacy of ITN and the proportion of children sleeping under an ITN. What the models do not accommodate are the simultaneous effects of increasing ITN coverage and declining access to effective medicines when children are febrile. In areas of high parasite transmission these combined effects may cancel, or increase severe complications leading to hospitalization or death.

Given the retrospective nature of hospital admissions to define historical trends there are a number of important caveats. First, inadequacies in routine hospital information systems plague many countries in sub-Saharan Africa and it remains difficult to identify hospitals with complete records covering 10 complete years. We have attempted to locate hospitals in representative malaria ecological settings that did have reasonable recording systems since 1999 and were therefore purposive rather than random in our selection of hospital sites. However, any bias would have favored better performing hospitals more likely to disprove any notion of increasing malaria trends and there was no prior expectation that admissions would increase. Second, despite a rapid increase in donor financing of malaria control in Uganda since 2005 there has not been an equivalent investment in monitoring the impact on transmission through routine parasitological or entomological surveillance. Entomological inoculation rates were high in almost all the sites investigated during the late 1990s [15] and childhood parasite prevalence was high at the end of the surveillance period in 2009 (Table 1) offering only a suggestion, rather than an empirical description that there has been little change in transmission. Third, because of a lack of longitudinal, high resolution data on the coverage of ITN, IRS or use of effective medicines to treat fever any attempt to attribute admission impact to changing intervention coverage through a plausibility framework is weakened by inadequate spatial and temporally resolved data. Finally, intriguing observations, whether they be declining or increasing disease incidence raise a number of important questions which unfortunately we and many others are unable to address retrospectively including the relative impacts of first line drug effectiveness with time, the changing patterns of alternative uses of clinical care providers in the community, the precise changing boundaries of catchment populations to hospitals within an area, patterns of resistance and tolerance to insecticides by dominant vector populations and many other factors that contribute to epidemiological transitions.

The problems facing analysis of available data on malaria trends in Africa are generic and not specific to the study presented here. However there is a concern that a more realistic assessment of the public health impact of scaled malaria control is constrained by the selective nature of reporting only 'good news' stories by donor agencies. Despite the limitations of historical reviews a more systematic country-level meta-analysis of available data is required to provide a more comprehensive understanding of the epidemiological transition in Africa and notably from areas that have the highest transmission intensity. These reviews should begin to explore the wealth of national data on intervention coverage, mortality and financing but must be undertaken subnationally to reflect the diversity of malaria risks common to most countries. Linkage to combined intervention access is non-trivial but with more data in time and space a clearer, less simplistic model of attribution might be possible. Without detailed observational epidemiology claims of success and the platform to decide upon future investments in control and new disease-prevention tools will remain ill informed.

## Conclusions

Our analysis of the changes in malaria admissions across Uganda show that the malaria transition witnessed in some parts of Africa is not true of all of Africa. Uganda is a long way from universal coverage of effective interventions, including ITN and access to effective treatments. In areas where transmission intensity is high, disease burdens remain high and most fevers will be associated with malaria infection and ensuring more than 80% coverage of interventions for prevention and prompt treatment is critical. In these areas modest increases in approaches to reduce infection risks or treatment of suspected cases we suggest will not impact on disease burdens to the same extent as proposed by the LiST models [61,67] in areas of less intense transmission and malaria morbidity and mortality is likely to continue to rise.

## Competing interests

RWS has received funding from Novartis for chairing meetings of national control programs in Africa and has received a research grant from Pfizer. All other authors have no conflicts of interest.

## Authors' contributions

EAO assembled all the hospital data, developed the analytical models and wrote the manuscript; DB and GM assisted in the primary assembly of the hospital data at each of the five hospitals; AM was the study coordinator in Uganda responsible for ensuring smooth running of the study and reviewed the manuscript; AOT was involved in the initial set up of the study and contributed to earlier drafts of the manuscript; RWS was responsible for the conception and its overall scientific management, analysis, interpretation and preparation of the final manuscript. All the authors read and approved the final manuscript.

## Pre-publication history

The pre-publication history for this paper can be accessed here:

http://www.biomedcentral.com/1741-7015/9/37/prepub
